# Combined influence of physical activity and C-reactive protein to albumin ratio on mortality among older cancer survivors in the United States: a prospective cohort study

**DOI:** 10.1186/s11556-024-00361-8

**Published:** 2024-10-02

**Authors:** Xiaoqin An, Jingyi Li, Yuan Li, Huanxian Liu, Junjun Bai, Qinxiang Guo, Baoping Jiao

**Affiliations:** 1 Department of Respiratory Medicine, Shanxi Province Cancer Hospital/ Shanxi Hospital Affiliated to Cancer Hospital, Chinese Academy of Medical Sciences/Cancer Hospital Affiliated to Shanxi Medical University, Taiyuan City, Shanxi Province China; 2 Department of Gastroenterology, Shanxi Province Cancer Hospital/ Shanxi Hospital Affiliated to Cancer Hospital, Chinese Academy of Medical Sciences/Cancer Hospital Affiliated to Shanxi Medical University, Taiyuan City, Shanxi Province China; 3 Department of Neurology, Chinese PLA General Hospital, Beijing, China; 4 Department of Thoracic Surgery, Shanxi Province Cancer Hospital/ Shanxi Hospital Affiliated to Cancer Hospital, Chinese Academy of Medical Sciences/Cancer Hospital Affiliated to Shanxi Medical University, Taiyuan City, Shanxi Province China; 5 Department of Hepatobiliary Pancreatic and Gastric Surgery, Shanxi Province Cancer Hospital/ Shanxi Hospital Affiliated to Cancer Hospital, Chinese Academy of Medical Sciences/Cancer Hospital Affiliated to Shanxi Medical University, Taiyuan City, Shanxi Province China

**Keywords:** Physical activity, C-reactive protein-to-albumin ratio, Older cancer survivals, Mortality, NHANES

## Abstract

**Background:**

Although a high C-reactive protein-to-albumin ratio (CAR) is believed to increase mortality risk, the association between the physical activity (PA), CAR, and mortality among cancer survivors has not been investigated. This study aimed to examine this association among cancer survivors in the United States.

**Methods:**

This cohort study used data from the National Health and Nutrition Examination Survey from 1999 to 2010. PA was self-reported using the Global Physical Activity Questionnaire, and C-reactive protein and albumin levels were obtained from laboratory data files. Mortality data were obtained by linkage of the cohort database to the National Death Index as of December 31, 2019. The analysis was conducted from November 1 to December 31, 2023. We used Cox proportional hazards multivariable regression to assess hazard ratios (HRs) and 95% confidence interval (CIs) for total and cancer-specific mortality risks attributable to PA and CAR.

**Results:**

Among 2,232 cancer survivors, 325 (14.6%) reported no PA with a high CAR. During a follow-up of up to 20.75 years (median, 12.3 years; 27,453 person-years), 1,174 deaths occurred (cancer, 335; other, 839). A high CAR was observed to be consistently associated with the highest risks of total (HR, 1.59; 95% CI, 1.37–1.85) and cancer-specific (HR, 2.06; 95% CI, 1.55–2.73) mortality compared with a low CAR in a series of adjusted models. Multivariable models showed that PA was associated with a lower risk of all-cause (HR, 0.60; 95% CI, 0.52–0.69) and cancer-specific (HR, 0.64; 95% CI, 0.49–0.84) mortality compared with no PA. In the joint analyses, survivors with PA ≥ 600 metabolic equivalent min/wk and a low CAR were more likely to reduce the risk of total (HR, 0.41; 95% CI, 0.32–0.51) and cancer-specific (HR, 0.32; 95% CI, 0.20–0.50) mortality by 59% and 68% compared with those with no PA and a high CAR.

**Conclusion:**

The pairing of adequate PA and a low CAR was significantly associated with reduced all-cause and cancer-related mortality risks.

**Supplementary Information:**

The online version contains supplementary material available at 10.1186/s11556-024-00361-8.

## Introduction

The number of cancer survivors is increasing rapidly worldwide [1]. By 1 January 2022, there will be more than 18 million cancer survivors in the United States, with 47% living more than 10 years; 18% living more than 20 years; and 67% aged 65 and older [2]. Medical advances have reduced the overall cancer death rate by approximately 32% over the past 30 years, and the overall 5-year survival rate for all cancers in the United States is approximately 68% [3, 4]. Many cancer survivors have complex physical conditions such as malnutrition, inflammation, and physical inactivity, which may contribute to the risk of recurrence and affect survival rates after cancer detection [5]. There are numerous modifiable factors for cancer survivors, including physical activity, dietary intake, smoking, alcohol consumption, and weight control [3]. Therefore, it is important to identify modifiable factors that can improve the long-term outcomes of cancer survivors.

C-reactive protein (CRP) and albumin are generated by the liver and are considered markers of inflammation and nutritional reserve, respectively [6–8]. The CRP-to-albumin ratio (CAR) is a new marker based on inflammation and nutritional status. CAR has been identified as a prognostic marker for infections, rheumatic diseases, malignant tumors, and critical conditions [9–12]. As a modifiable biomarker, CAR can be influenced by lifestyle factors. Prior studies have demonstrated that enhancing nutritional intake, particularly through a balanced breakfast, can lead to significant improvements in albumin levels among elderly patients. Similarly, smoking cessation has been correlated with a gradual reduction in CRP levels [13, 14]. However, research on the association between CAR and mortality in cancer survivors is lacking, with current research primarily focusing on the prognosis of critically ill patients and treatment response in cancer patients.

Physical activity (PA) has long been recognized for improving health in chronic conditions, including obesity, type 2 diabetes, neurodegenerative disorders, cardiovascular diseases, and cancer [15, 16]. Evidence suggests that PA is linked to the enhanced survival of patients with various types of cancer [17]. However, it remains unclear whether survivors of all cancer types benefit from PA because the levels are significantly low among cancer survivors [18]. Previous studies on PA have primarily focused on obesity-related cancers [19, 20].

A higher CAR in cancer survivors is associated with poorer immune and nutritional status, which in turn is linked to an increased risk of mortality [9–12]. Previous epidemiological studies have demonstrated that exercise can enhance the physical functioning of cancer survivors, reinforce their immune system, and mitigate negative emotions such as anxiety and depression [17]. The hypothesis was that physical activity could reduce CRP levels and improve albumin levels, thereby exerting a modulating effect on the relationship between CAR and mortality. Therefore, this study aimed to investigate the independent and combined associations of the CAR and PA on long-term mortality among cancer survivors.

## Methods

This prospective cohort study was based on a nationally representative sample from the United States National Center for Health Statistics National Health and Nutrition Examination Survey (NHANES). This survey utilizes stratified, multistage probability cluster sampling, and has been performed in 2-year cycles since 1999 to manage the health and nutritional status of non-institutionalized populations in the United States [21, 22]. This survey has been performed in 2-year cycles since 1999 to manage the health and nutritional status of the United States population. Informed consent was obtained from all participants, and all NHANES protocols were approved by the Ethics Review Board of the National Center for Health Statistics. This modeling investigation was exempt from review because it used published, de-identified datasets with no personal information. This study followed the Strengthening the Reporting of Observational Studies in Epidemiology (STROBE) Statement declaration.

### Study population

Each participant was interviewed and asked to complete a series of physical examinations and laboratory tests at a mobile examination center. This study analyzed data on sociodemographic traits, lifestyle factors, laboratory results, and medical history in adults aged 40 or older with available CRP, albumin, and PA data from 6 NHANES cycles from 1999 to 2010.

### Cancer diagnosis

Information on the cancer diagnosis and type was obtained through face-to-face interviews. Interviewees were asked, “Have you ever been told by a doctor or other health professional that you have cancer or a malignant tumor of any kind?” Individuals who replied yes were defined as cancer survivors and were asked, “What type of cancer was it?” Cancer types were further grouped into obesity-related and non-obesity-related cancers, as research on PA has mainly focused on obesity-related cancers [19, 20]. Cancers of the breast, colon, rectum, esophagus, gallbladder, kidney, liver, ovary, pancreas, stomach, and uterus were classified as obesity-related cancers, whereas other cancers were classified as non-obesity-related cancers [23, 24].

### Calculation formula and grouping of CAR

The CAR was calculated as CRP divided by the albumin level. Tertiles of CAR were categorized as low (< 0.034), medium (0.034–0.102), and high (≥ 0.102) because of the lack of established CAR cut-off values [25, 26].

### PA assessment

Data were collected using the Global Physical Activity Questionnaire created by the World Health Organization (WHO) to assess different domains of PA, such as leisure-time PA, occupation, and transportation PA [27]. PA was converted to metabolic equivalent (MET) minutes of moderate-to-vigorous PA per week in accordance with the WHO analysis guidelines. The MET values for each type of exercise were obtained from the NHANES, and PA was calculated using the following formula: PA (MET-min/week) = MET × weekly frequency × duration of each PA [28]. According to the 2018 Physical Activity Guidelines for Americans, adults should engage in moderate-intensity PA for 150 min per week (equivalent to 600 MET min/wk) or vigorous-intensity activity for 75 min per week [15]. Based on the MET calculation formula and the PA requirements in the guidelines, we made the following classification of PA. Participants were classified as inactive (PA = 0), insufficiently active (0 < PA < 600 MET-min/wk), and active (PA ≥ 600 MET-min/wk), respectively.

### Mortality assessment

The National Center for Health Statistics provided mortality data linked to the National Death Index through December 31, 2019, using the International Statistical Classification of Diseases and Related Health Problems, Tenth Revision (ICD-10) to record the underlying cause of death. Cancer mortality was classified as death caused by malignant neoplasms (ICD-10, codes C00–C97). The duration of follow-up was defined as the interval in months from the interview date to the date of death or December 31, 2019, for participants who did not experience an event.

### Covariate assessment

Based on previous research and expert clinical opinions, potential variables that could confound or modify the results were identified [19]. These variables include age, sex (man or woman), race or ethnicity (non-Hispanic White, non-Hispanic Black, Mexican American, other races [including multiracial and other Hispanic]), educational attainment (less than high school, high school or equivalent, and above high school), marital status (married, living with a partner, widowed, divorced, separated, or never married), poverty-to-income ratio (total family income divided by the poverty threshold; < 1.3, 1.3 to ≤ 3.5, ≥ 3.5) [29], body mass index (BMI; calculated based on the ratio of weight in kilograms to height in meters squared) categorized into three groups (< 25, 25.0–29.9, ≥ 30), smoking status (current, former, or never), alcohol use (never, former, or current), and the Healthy Eating Index-2015 [30] (HEI-2015; derived from the participant’s 24-h dietary recall interview). The HEI-2015 measures the quality of an individual’s overall diet, with scores ranging from 0 to 100 (worst to best). Hypertension was either self-reported by participants who had received a diagnosis from a health professional or determined by the NHANES-measured blood pressure (≥ 130 mm Hg [systolic] or ≥ 80 mm Hg [diastolic]). A history of cardiovascular disease and diabetes was self-reported by participants who had received either or both of these diagnoses from a health professional or were determined by a prescription history for medications used to treat these conditions.

### Statistical analysis

This study involved a secondary analysis of publicly available datasets, and all analyses were conducted in accordance with the NHANES analytic guidelines. Continuous variables are presented as mean ± standard deviation (SD) or median (interquartile range), while categorical variables are presented as frequencies or percentages. One-way analysis of variance was used to test statistical differences among the CAR groups for continuous variables and the chi-square test for categorical variables in the baseline characteristics analysis. Additionally, some variables (poverty-to-income ratio, BMI, and HEI-2015) had missing data, and the K-nearest neighbors' method was used to interpolate the missing values. Statistical tests were two-sided, and statistical significance was set at *P* < 0.05.

Multivariable Cox proportional hazards regression models were used to estimate hazard ratios (HR) and 95% confidence intervals (CI) for the associations of CAR and PA with overall and cancer-specific mortality, respectively. The final-stage multivariable models were adjusted for age, sex, race, ethnicity, educational attainment, family poverty income ratio, BMI, smoking status, alcohol use, HEI-2015 score, and health factors (hypertension, history of diabetes, and cardiovascular disease). To examine joint associations, participants were classified according to their CAR and PA levels (Low CAR + Inactive PA; Low CAR + Insufficiently active PA; Low CAR + Active PA; Medium CAR + Inactive PA; Medium CAR + Insufficiently active PA; Medium CAR + Active PA; High CAR + Inactive PA; High CAR + Insufficiently active PA; High CAR + Active PA), and mortality risks were estimated using multivariable Cox proportional hazards regression models adjusted for the same set of covariates. All analyses were conducted for the overall population, as well as for women, men, and survivors of obesity- and non-obesity-related cancers. Sensitivity analyses were conducted by excluding deaths during the first 2-year follow-up to reduce the probability of reverse causation [31]*.* Finally, the restricted cubic splines with Cox proportional hazard models were used to depict the linearity/nonlinearity associations between InCAR and HRs of all-cause mortality.

R software (version 4.3.2; R Foundation for Statistical Computing; http://www.Rproject.org) and Free Statistics software (version 1.9; Beijing Free Clinical Medical Technology Co., Ltd.) were used for the analysis. Data analysis was performed from October 1 to December 30, 2023.

## Results

### Study population

Of the 32,464 participants aged ≥ 20 years, 30,232 were excluded because they had no history of cancer (*n* = 29,470), were less than 40 years of age (*n* = 217), had missing data for CRP (*n* = 407), albumin (*n* = 18), smoking status (*n* = 2), alcohol use (*n* = 119), and hypertension (*n* = 1). The final analysis included 2,232 participants (e Fig. 2).

### Baseline characteristics

Of the 2,232 cancer survivors (mean [SD] age, 68.7 [11.6] years; 50.3% men) in the study cohort, participants were categorized into three groups based on the tertiles of CAR: low (*n* = 739), medium (*n* = 747), and high (*n* = 746). Of these, 1,727 (77.4%) were non-Hispanic White, 255 (11.4%) were non-Hispanic Black, 147 (6.6%) were Mexican American, and 103 (4.6%) were individuals of “other” race or ethnicity, including multiracial and other Hispanic (Table 1). The number of cancer cases according to type and sex is shown in eTable 1 in the Supplement. Only 38.0% of the cancer survivors were physically active (PA ≥ 600 MET min/wk), while 34.0% reported no PA the previous week. Remarkably, 325 (14.6%) cancer survivors reported being physically inactive, with a high CAR (eTable 2).

**Table 1:** Baseline characteristics based on car tertiles in the national health and nutrition examination survey, 1999–2010

Characteristic	All	No. of participants by CAR ^a^			*P* value
		Low	Medium	High	
Overall	2232	739	747	746	
Age (year),Mean (SD)	68.7 (11.6)	68.4(11.6)	69.7(11.3)	67.9(11.7)	.012
Sex, n (%)					< .001
Male	1122 (50.3)	423 (57.2)	369 (49.4)	330 (44.2)	
Female	1110 (49.7)	316 (42.8)	378 (50.6)	416 (55.8)	
Race and ethnicity, n (%)					< .001
Non-Hispanic White	1727 (77.4)	597 (80.8)	596 (79.8)	534 (71.6)	
Non-Hispanic Black	255 (11.4)	64 (8.7)	73 (9.8)	118 (15.8)	
Mexican American	147 (6.6)	44 (6.0)	46 (6.2)	57 (7.6)	
Other ^b^	103 (4.6)	34 (4.6)	32 (4.3)	37 (5.0)	
Marital status, n (%)					.008
Married or living with a partner	1413 (63.3)	495 (67.0)	476 (63.7)	442 (59.2)	
Living alone	819 (36.7)	244 (33.0)	271 (36.3)	304 (40.8)	
Family poverty income ratio, n (%)					< .001
< 1.3	435 (19.5)	98 (13.3)	151 (20.2)	186 (24.9)	
1.3 to 3.5	991 (44.4)	310 (41.9)	333 (44.6)	348 (46.6)	
≥ 3.5	806 (36.1)	331 (44.8)	263 (35.2)	212 (28.4)	
Educational level, n (%)					< .001
Less than high school	578 (25.9)	160 (21.7)	189 (25.3)	229 (30.7)	
High school or equivalent	546 (24.5)	173 (23.4)	190 (25.4)	183 (24.5)	
Above high school	1108 (49.6)	406 (54.9)	368 (49.3)	334 (44.8)	
Smoking status, n (%)					.013
Never	956 (42.8)	347 (47.0)	308 (41.2)	301 (40.3)	
Former	988 (44.3)	317 (42.9)	340 (45.5)	331 (44.4)	
Current	288 (12.9)	75 (10.1)	99 (13.3)	114 (15.3)	
Alcohol drinking status, n (%)					< .001
Never	327 (14.7)	90 (12.2)	98 (13.1)	139 (18.6)	
Former	665 (29.8)	178 (24.1)	226 (30.3)	261 (35.0)	
Current	1240 (55.6)	471 (63.7)	423 (56.6)	346 (46.4)	
HEI-2015, Mean (SD)	55.9(12.9)	58.0 (13.1)	55.6(12.9)	54.3(12.4)	< .001
Body mass index (kg/m2), n (%)					< .001
< 25	650 (29.1)	309 (41.8)	190 (25.4)	151 (20.2)	
25 to 30	862 (38.6)	300 (40.6)	319 (42.7)	243 (32.6)	
≥ 30	720 (32.3)	130 (17.6)	238 (31.9)	352 (47.2)	
Diabetes, n (%)					< .001
No	1678 (75.2)	581 (78.6)	577 (77.2)	520 (69.7)	
Yes	554 (24.8)	158 (21.4)	170 (22.8)	226 (30.3)	
Cardiovascular disease, n (%)					< .001
No	1637 (73.3)	568 (76.9)	559 (74.8)	510 (68.4)	
Yes	595 (26.7)	171 (23.1)	188 (25.2)	236 (31.6)	
Hypertension, n (%)					.001
No	749 (33.6)	284 (38.4)	245 (32.8)	220 (29.5)	
Yes	1483 (66.4)	455 (61.6)	502 (67.2)	526 (70.5)	
Physical Activity, (MET-min/week)					< .001
None (inactive)	759 (34.0)	186 (25.2)	248 (33.2)	325 (43.6)	
< 600 (insufficiently active)	626 (28.0)	223 (30.2)	223 (29.9)	180 (24.1)	
≥ 600 (sufficiently active)	847 (37.9)	330 (44.7)	276 (36.9)	241 (32.3)	

*Abbreviations: CAR* C-reactive protein-to-albumin ratio, *SD* Standard deviation, *HEI-2015* Healthy Eating Index-2015, *BMI* Body mass index (calculated as weight in kilograms divided by height in meters squared); MET, the metabolic equivalent

^a^Grouped by tertiles into low (< 0.034), medium (0.034–0.102), high (> 0.102)

^b^Other includes multiple races and other Hispanics

#### Association between single CAR or PA and mortality

During the 20.75 years of follow-up (median, 12.3 years; 27,453 person-years), 1,174 deaths occurred (cancer, 335; other, 839). A high CAR was observed to be consistently associated with the highest risks of total (HR, 1.59; 95% CI, 1.37–1.85) and cancer-specific (HR, 2.06; 95% CI, 1.55–2.73) mortality compared with a low CAR in a series of adjusted models. In multivariable models, being physically active was associated with a lower risk of total (HR, 0.60; 95% CI, 0.52–0.69) and cancer-specific (HR, 0.64; 95% CI, 0.49–0.84) mortality compared with being physically inactive. Even insufficient PA reduced the risk of all-cause death and cancer-specific death by 35% and 33% compared with inactivity (Table 2). These findings were consistent between women and men and between those diagnosed with obesity- and non-obesity-related cancers (eTable 3).

**Table 2:** Association between CAR and Physical Activity with all-cause, cancer mortality among US cancer survivors aged 40 years or older, NHANES, 1999 to 2010 in multiple regression model

Mortality Outcome	Deaths/no	Non-adjusted Model		Model I^a^		Model II^b^	
		HR (95% CI)	*P*-value	HR (95% CI)	*P*-value	HR (95% CI)	*P*-value
**All Causes**							
CAR							
Low	329/739	1 [Reference]		1 [Reference]		1 [Reference]	
Medium	407/747	1.30 (1.12 ~ 1.50)	< .001	1.22 (1.06 ~ 1.42)	.007	1.20 (1.04 ~ 1.40)	.014
High	438/746	1.53 (1.33 ~ 1.77)	< .001	1.64 (1.42 ~ 1.90)	< .001	1.59 (1.37 ~ 1.85)	< .001
Per SD increase	NA	1.16 (1.12 ~ 1.22)	< .001	1.14 (1.09 ~ 1.18)	< .001	1.12 (1.08 ~ 1.16)	< .001
Physical Activity(MET-min/week)							
None(inactive)	508/759	1 [Reference]		1 [Reference]		1 [Reference]	
< 600 (insufficiently active)	318/626	0.52 (0.45 ~ 0.60)	< .001	0.63 (0.54 ~ 0.73)	< 0.001	0.65 (0.56 ~ 0.75)	< .001
≥ 600 (sufficiently active)	348/847	0.47 (0.41 ~ 0.54)	< .001	0.55 (0.47 ~ 0.63)	< 0.001	0.60 (0.52 ~ 0.69)	< .001
**Cancer**							
CAR							
Low	83/739	1 [Reference]		1 [Reference]		1 [Reference]	
Medium	109/747	1.37 (1.03 ~ 1.82)	.032	1.34 (1.01 ~ 1.79)	.043	1.27 (0.95 ~ 1.7)	.105
High	143/746	1.96 (1.50 ~ 2.57)	< .001	2.07 (1.57 ~ 2.73)	< .001	2.06 (1.55 ~ 2.73)	< .001
Per SD increase	NA	1.21 (1.13 ~ 1.28)	< .001	1.17 (1.1 ~ 1.25)	< .001	1.18 (1.11 ~ 1.26)	< .001
Physical Activity(MET-min/week)							
None(inactive)	137/759	1 [Reference]		1 [Reference]		1 [Reference]	
< 600 (insufficiently active)	90/626	0.59 (0.45 ~ 0.77)	< .001	0.66 (0.5 ~ 0.88)	.004	0.67 (0.51 ~ 0.88)	.005
≥ 600 (sufficiently active)	108/847	0.56 (0.43 ~ 0.72)	< .001	0.61 (0.47 ~ 0.79)	< .001	0.64 (0.49 ~ 0.84)	.001

*Abbreviations: CAR* C-reactive protein-to-albumin ratio, *US* the United States, *NHANES* National Health and Nutrition Examination Survey, *HR* Hazard ratio, *CI* Confidence interval, *MET* the metabolic equivalent

^a^ Multivariable model adjusts for age, sex, race (Non-Hispanic White, Non-Hispanic Black, Mexican American, Other Race—including multiracial and other Hispanic), Marital status (Married or living with a partner, Living alone), educational attainment (less than high school, high school or equivalent, and above high school), family poverty income ratio (< 1.30, 1.30–3.49, or ≥ 3.5)

^b^ Additionally adjusted for BMI (calculated as weight in kilograms divided by height in meters squared), smoking status (never, former, current), alcohol use (never, former, current), Healthy Eating Index-2015, hypertension (yes/no), history of diabetes (yes/no), cardiovascular disease (yes/no)

### Joint association between CAR and PA with mortality

For people who were insufficiently active or inactive, CAR was linked to a higher risk of overall (HR per SD increase, 1.16 [95% CI, 1.10–1.22]) and cancer (HR per SD increase, 1.24 [95% CI, 1.15–1.34]) mortality, increasing in a dose–response manner (Table 3 and eFigure 1).

**Table 3:** Association of CAR With All-Cause, Cancer Mortality Among US Cancer Survivors ≥ 40 years Stratified by PA Level

Mortality Outcome	Hazard Ratio (95% CI)^a^	
	PA ≥ 600 MET minutes/week (Physically Active)	PA < 600 MET minutes/week (Inactive/Insufficient Active)
**All-Cause**		
CAR		
Low	1 [reference]	1 [reference]
Medium	1.11 (0.86 ~ 1.44)	1.24 (1.03 ~ 1.49)
High	1.49 (1.14 ~ 1.95)	1.64 (1.37 ~ 1.97)
per SD increase	1.04 (0.98 to 1.11)	1.16 (1.10 ~ 1.22)
**Cancer**		
CAR		
Low	1 [reference]	1 [reference]
Medium	1.64 (1.01 ~ 2.67)	1.09 (0.75 ~ 1.57)
High	2.44 (1.48 ~ 4.02)	1.89 (1.33 ~ 2.68)
per SD increase	1.04 (0.93 to 1.17)	1.24 (1.15 ~ 1.34)

*Abbreviations: CAR* C-reactive protein-to-albumin ratio, *US* the United States, *PA* Physical Activity, *CI* Confidence interval, *MET* The metabolic equivalent

^a^ Adjusted for age (years), sex (male or female), race/ethnicity (non-Hispanic white, non-Hispanic black, Hispanic, and other), marital status (married or living with partner, widowed or divorced or separated or never married), education attainment (less than high school, high school graduate, above high school), family poverty ratio (< 1.30, 1.30–3.49, or ≥ 3.5), body mass index (BMI; calculated as weight in kilograms divided by height in meters squared) (< 25, 25–29.9, and ≥ 30), smoking status (never, former, and current), alcohol use (never, former, and current), Healthy Eating Index-2015, hypertension (yes or no), history of diabetes (yes or no), history of CVD (yes or no)

**Fig. 1:**
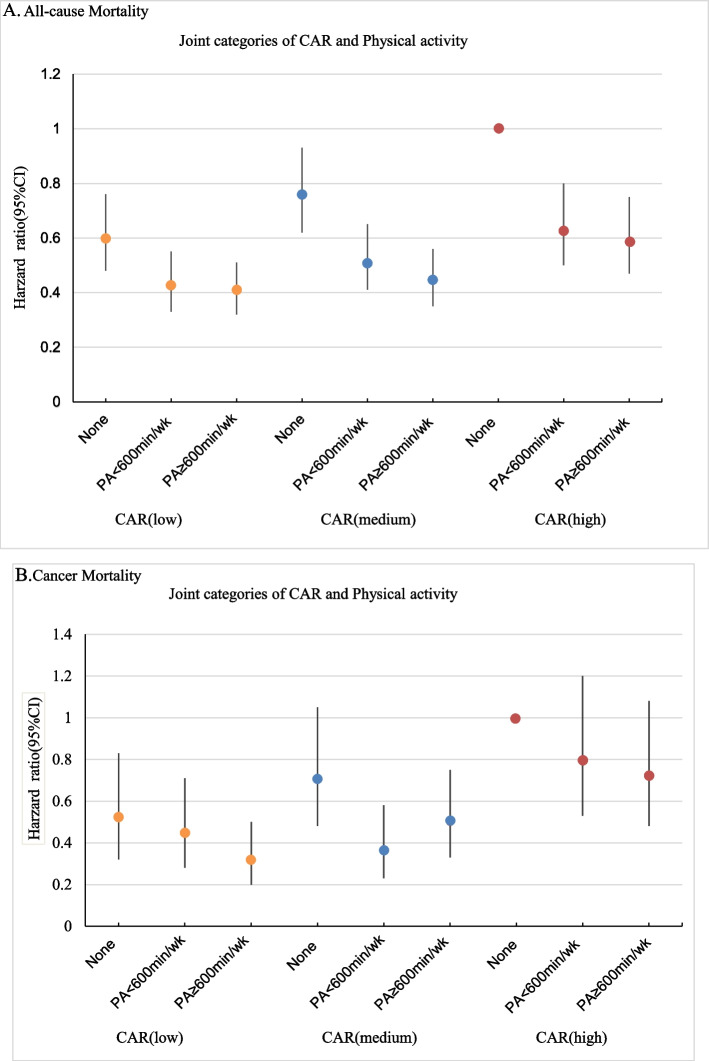
Joint association of C-reactive protein to albumin ratio and Physical activity with all-cause and cancer mortality among US cancer survivors age 40 years or older, NHANES, 1999 to 2010

The combined effects of CAR and PA on all-cause mortality were further analyzed by grouping CAR tertiles and PA levels into a 9-category variable to represent joint exposures. Among the various combinations, cancer survivors with high PA and lower CAR showed favorable associations with the risk of all-cause and cancer-specific mortality. Specifically, survivors with PA ≥ 600 MET min/wk and a low CAR were more likely to reduce the risk of all-cause (HR, 0.41; 95% CI, 0.32–0.51) and cancer-specific (HR, 0.32; 95% CI, 0.20–0.50) mortality by 59% and 68% respectively, compared with those with no PA and a high CAR (Table 4 and Fig. 1). However, cancer survivors with a high CAR may not benefit from PA for cancer mortality, and these results were consistent across sexes and remained similar between survivors with obesity- and non-obesity-related cancer diagnoses (eTable 4). All results remained similar in the sensitivity analyses, excluding deaths during the first 2 years (eTables 5 and 6).

**Table 4:** Joint association between CAR and Physical Activity with all-cause and cancer mortality among US cancer survivors age 40 years or older, NHANES, 1999 to 2010

Mortality Outcome	Physical Activity (MET-min/week)	Death/No	Hazard ratio (95% CI)		
			Non-adjusted Model	Model I^a^	Model II^b^
**All-Cause**					
CAR (low)	None(inactive)	108/186	0.73 (0.58 ~ 0.92)	0.60 (0.48 ~ 0.76)	0.60 (0.48 ~ 0.76)
	< 600 (insufficiently active)	96/223	0.38 (0.30 ~ 0.49)	0.43 (0.34 ~ 0.55)	0.43 (0.33 ~ 0.55)
	≥ 600 (sufficiently active)	1125/330	0.39 (0.31 ~ 0.48)	0.37 (0.3 ~ 0.47)	0.41 (0.32 ~ 0.51)
CAR (medium)	None(inactive)	171/248	0.95 (0.78 ~ 1.16)	0.78 (0.64 ~ 0.95)	0.76 (0.62 ~ 0.93)
	< 600 (insufficiently active)	121/223	0.50 (0.40 ~ 0.63)	0.49 (0.39 ~ 0.61)	0.51 (0.41 ~ 0.65)
	≥ 600 (sufficiently active)	115/276	0.43 (0.35 ~ 0.54)	0.42 (0.34 ~ 0.54)	0.45 (0.35 ~ 0.56)
CAR (high)	None(inactive)	229/325	1 [Reference]	1 [Reference]	1 [Reference]
	< 600 (insufficiently active)	101/180	0.57 (0.45 ~ 0.72)	0.63 (0.50 ~ 0.80)	0.63 (0.50 ~ 0.80)
	≥ 600 (sufficiently active)	108/241	0.49 (0.39 ~ 0.62)	0.55 (0.44 ~ 0.70)	0.59 (0.47 ~ 0.75)
**Cancer**					
CAR (low)	None(inactive)	24/186	0.55 (0.34 ~ 0.87)	0.49 (0.31 ~ 0.78)	0.52 (0.32 ~ 0.83)
	< 600 (insufficiently active)	29/223	0.42 (0.27 ~ 0.65)	0.45 (0.29 ~ 0.71)	0.45 (0.28 ~ 0.71)
	≥ 600 (sufficiently active)	30/330	0.32 (0.21 ~ 0.49)	0.31 (0.20 ~ 0.48)	0.32 (0.20 ~ 0.50)
CAR (medium)	None(inactive)	45/248	0.83 (0.57 ~ 1.21)	0.76 (0.52 ~ 1.12)	0.71 (0.48 ~ 1.05)
	< 600 (insufficiently active)	25/223	0.37 (0.24 ~ 0.59)	0.38 (0.24 ~ 0.6)	0.36 (0.23 ~ 0.58)
	≥ 600 (sufficiently active)	39/276	0.50 (0.34 ~ 0.75)	0.49 (0.33 ~ 0.74)	0.50 (0.33 ~ 0.75)
CAR (high)	None(inactive)	68/325	1 [Reference]	1 [Reference]	1 [Reference]
	< 600 (insufficiently active)	36/180	0.72 (0.48 ~ 1.08)	0.77 (0.51 ~ 1.16)	0.79 (0.53 ~ 1.20)
	≥ 600 (sufficiently active)	39/241	0.61 (0.41 ~ 0.91)	0.69 (0.46 ~ 1.03)	0.72 (0.48 ~ 1.08)

*Abbreviations: CAR* C-reactive protein-to-albumin ratio; *US* the United States, *NHANES* National Health and Nutrition Examination Survey, *HR* Hazard ratio, *CI* Confidence interval, *MET* the metabolic equivalent

^a^Multivariable model adjusts for age, sex, race (Non-Hispanic White, Non-Hispanic Black, Mexican American, Other Race—including multiracial and other Hispanic), Marital status (Married or living with a partner, Living alone), educational attainment (less than high school, high school or equivalent, and above high school), family poverty income ratio (< 1.30, 1.30–3.49, or ≥ 3.5)

^b^Additionally adjusted for BMI (calculated as weight in kilograms divided by height in meters squared), smoking status (never, former, current), alcohol use (never, former, current), Healthy Eating Index-2015, hypertension (yes/no), history of diabetes (yes/no), cardiovascular disease (yes/no)

## Discussion

In this prospective cohort study, an increased CAR and decreased PA were associated with a higher risk of all-cause and cancer-related mortality. Cancer survivors with a low CAR and adequate PA had the lowest risk of mortality compared with those with a high CAR and inadequate PA. These findings emphasize the importance of risk management through PA, particularly in patients with low and moderate CAR.

Previous studies have shown that cancer patients are susceptible to concurrent malnutrition, inflammation due to immune deficiencies, and metabolic disorders [32, 33]. The CAR can help to elucidate inflammation and nutritional status. Several epidemiological studies have described a positive association between CAR, early recurrence, and poor survival among certain cancers [9, 34–38]. In a retrospective study among Japanese participants, CAR ≥ 0.028 (HR 2.30 and 1.58, respectively) was found to predict early and late mortality in early gastric cancers [39]. In this study, we discovered notable links between a high CAR and higher total and cancer-specific mortality in participants categorized according to various PA levels, utilizing continuous measurements and CAR tertiles. Therefore, when screening high-risk populations of cancer survivors, we should specifically focus on individuals with simultaneous increases in CRP levels and decreases in albumin levels.

Exercise boosts the immune system’s ability to detect and destroy tumors [40]. Research has shown that physical activity significantly reduces the risk of mortality from breast and colon cancers [41, 42]. Furthermore, a clinical trial has indicated that exercise may boost the infiltration of natural killer cells, leading to improved survival rates in patients with melanoma [43]. While clinical evidence for other cancer types is scarce, preclinical studies in animal models suggest that regular exercise can provide beneficial preventive and therapeutic effects against various cancers, including prostate cancer, lung cancer, and chronic lymphocytic leukemia [44]. In our current study, we found significant links between PA and lower overall and cancer-related mortality among cancer survivors.

To our knowledge, this is the most extensive prospective cohort study to date examining the separate and combined health effects of PA and CAR on the mortality of cancer survivors. Our joint analyses revealed that PA could lower the risk of total mortality by 41% in the high CAR subgroup. However, the specific mechanisms driving the potential interactions between PA and the CAR remain unclear. Previous research suggests that PA may decrease the risk of various adverse health outcomes in cancer survivors, in part because of its anti-inflammatory effect on the inflammatory process [45, 46]. Similar to our findings, a large prospective study involving 56,282 participants from China and the United States found that PA may reduce the link between the systemic immune inflammation index and overall mortality in middle-aged and older populations [47]. A cohort study based on high-sensitivity CPR, PA, and mortality found that being physically active lowered the risk of death in South Korean adults with high CRP levels [48]. These findings highlight the importance of PA in preventing all-cause and cause-specific mortality related to chronic inflammation. Further investigation of the biological mechanisms of CAR and PA associated with mortality is needed from population- and animal-based studies.

### Strengths and limitations

One of the main strengths of our study was the use of a nationally representative sample of adults in the United States, which enhanced the reliability of the results. Additionally, the comprehensive and high-quality data collected enabled us to account for well-known confounding factors, including socioeconomic status, lifestyle, and health conditions.

Some limitations, however, should be noted. First, a single routine blood measurement and the CAR at baseline may not accurately reflect the long-term status of chronic inflammation over the follow-up period, potentially leading to an underestimation of the association between CAR and mortality. Similarly, measuring PA only at baseline does not capture changes in PA that are likely to occur over time and may therefore be an inadequate measure of exposure. The calculation of MET scores based on a questionnaire may be susceptible to significant limitations, particularly given the inherent risk of bias associated with self-disclosure, which could lead to inaccurate results. Secondly, our results might have been influenced by reverse causation or residual confounding. However, the associations persisted even after excluding participants who died during the first 2 years of follow-up, minimizing the possibility of reverse causation. Third, The process of converting continuous variables into categorical variables may result in the loss of information. For instance, the classification of PA into "Inactive", "Insufficiently active" and "Active" categories, while a relatively simple and readily understandable approach, may lead to the obfuscation of details on specific physical activity levels. A similar degree of caution is required in the interpretation of CAR categories and covariates such as poverty-to-income ratio. Further research may yield more profound insights by contemplating these variables as continuous or by employing more refined categorizations to accurately delineate their interrelationships with mortality in cancer survivors. Fourth, the observational nature of the study limits the ability to provide information on the biological mechanisms underlying the results. Further in vivo and in vitro studies are required to elucidate these mechanisms.

## Conclusions

A high CAR can elevate the risk of death, which underscores the need to focus on individuals with high CRP and low albumin levels. The pairing of adequate PA and a low CAR was significantly associated with reduced all-cause and cancer-related mortality risks. By further comprehending these connections, public health professionals can create more effective interventions and policies to enhance the long-term health outcomes of cancer survivors.

## Supplementary Information

Supplementary Material 1

## Data Availability

These survey data are free and publicly available and can be downloaded directly from the NHANES website (http://www.cdc.gov/nchs/nhanes.htm) by users and researchers worldwide.

## Abbreviations

BMI: Body mass index
CI: Confidence interval
CAR: C-reactive protein-to-albumin ratio
CRP: C-reactive protein
HEI-2015: Healthy Eating Index-2015
HR: Hazard ratio
ICD-10: International Statistical Classification of Diseases and Related Health Problems, Tenth Revision
MET: Metabolic equivalent
NHANES: National Health and Nutrition Examination Survey
PA: Physical activity
SD: Standard deviation
WHO: World Health Organization
