# Supplementary material for: Combined influence of physical activity and C-reactive protein to albumin ratio on mortality among older cancer survivors in the United States: a prospective cohort study
Source: Eur Rev Aging Phys Act. 2024 Oct 2;21:26. doi: 10.1186/s11556-024-00361-8 (PMC11448037; doi:10.1186/s11556-024-00361-8)

**Supplemental Online Content**

**eTable 1.** Numbers of Cancer Survivors by Cancer Type and Sex, NHANES 1999-2010

**eTable 2.** Joint Prevalence of C-reactive protein to albumin ratio and Physical a

ctivity Level Among US Cancer Survivors ≥40 years, NHANES 1999-2010

**eFigure1.** Dose-Response Association Between InCAR and all-cause Mortality Among US Cancer Survivors

**eTable 3.** Association of CAR and Physical activity Level with All-Cause Mortality Among US Cancer Survivors ≥40 years by Sex and Obesity-Related Cancer, NHANES 1999-2010

**eTable 4.** Joint Association of CAR and Physical activity Level With All-Cause Mortality Among US Cancer Survivors ≥40 years By Sex and Obesity-Related Cancer, NHANES 1999-2010

**eTable 5.** Association between C-reactive protein to albumin ratio and risk of death in multiple regression model(sensitivity analysis, excluding deaths that occurred during the first 2-year follow-up,n=2093).

**eTable 6.**Joint association between C-reactive protein to albumin ratio and Physical activity with all-cause and cancer mortality among US cancer survivors age 40 years or older, NHANES, 1999 to 2010.(sensitivity analysis, excluding deaths that occurred during the first 2-year follow-up,n=2093).

**eFigure2.**  Flowchart of patient selection.

| **eTable 1. Numbers of Cancer Survivors by Cancer Type and Sex,**  **NHANES 1999-2010** | | | |
| --- | --- | --- | --- |
| **Cancer Type(%)** | **Numbers of Cancer Survivors** | | |
|  | **Male** | **Female** | **Total** |
|  | 1122 | 1110 | 2232 |
| Bladder | 45 (4) | 9 (0.8) | 54 ( 2.4) |
| Blood | 2 (0.2) | 0 (0) | 2 ( 0.1) |
| Bone | 7 (0.6) | 7 (0.6) | 14 ( 0.6) |
| Brain | 8 (0.7) | 2 (0.2) | 10 ( 0.4) |
| Breast | 0 (0) | 333 (30.1) | 333 (15.0) |
| Cervix (cervical) | 0 (0) | 111 (10) | 111 ( 5.0) |
| Colon | 87 (7.8) | 75 (6.8) | 162 ( 7.3) |
| Esophagus (esophageal) | 9 (0.8) | 2 (0.2) | 11 ( 0.5) |
| Gallbladder | 0 (0) | 1 (0.1) | 1 ( 0.0) |
| Kidney | 21 (1.9) | 16 (1.4) | 37 ( 1.7) |
| Larynx/ windpipe | 14 (1.3) | 0 (0) | 14 ( 0.6) |
| Leukemia | 16 (1.4) | 4 (0.4) | 20 ( 0.9) |
| Liver | 1 (0.1) | 3 (0.3) | 4 ( 0.2) |
| Lung | 33 (3) | 19 (1.7) | 52 ( 2.3) |
| Lymphoma/ Hodgkin's disease | 19 (1.7) | 16 (1.4) | 35 ( 1.6) |
| Melanoma | 64 (5.7) | 57 (5.1) | 121 ( 5.4) |
| Mouth/tongue/lip | 11 (1) | 1 (0.1) | 12 ( 0.5) |
| Ovary (ovarian) | 0 (0) | 46 (4.2) | 46 ( 2.1) |
| Pancreas (pancreatic) | 2 (0.2) | 0 (0) | 2 ( 0.1) |
| Prostate | 351 (31.4) | 0 (0) | 351 (15.8) |
| Rectum (rectal) | 5 (0.4) | 5 (0.5) | 10 ( 0.4) |
| Skin (non-melanoma) | 225 (20.1) | 160 (14.5) | 385 (17.3) |
| Skin (don't know what kind) | 118 (10.6) | 71 (6.4) | 189 ( 8.5) |
| Soft tissue (muscle or fat) | 2 (0.2) | 3 (0.3) | 5 ( 0.2) |
| Stomach | 6 (0.5) | 10 (0.9) | 16 ( 0.7) |
| Testis (testicular) | 11 (1) | 0 (0) | 11 ( 0.5) |
| Thyroid | 12 (1.1) | 24 (2.2) | 36 ( 1.6) |
| Uterus (uterine) | 0 (0) | 94 (8.5) | 94 ( 4.2) |
| Other | 48(4.3) | 38(3.4) | 863.9) |
| Missing | 4(0.35) | 3(0.27) | 7(0.31) |

| **eTable 2. Joint Prevalence of C-reactive protein to albumin ratio and Physical Activity Level Among US Cancer Survivors ≥40 years, NHANES 1999-2010** | | | | |
| --- | --- | --- | --- | --- |
|  | **C-reactive protein to albumin ratio** | | | |
| **Physical Activity Level** | Low | Medium | High | Total |
| No PA (Inactive) | 186（8.3） | 248（11.1） | 325（14.6） | 759（34.0） |
| 0-<600 MET minutes/week PA (Insufficient Active) | 223（10.0） | 223（10.0） | 180（8.1） | 626（28.0） |
| ≥600 MET minutes/week PA (Sufficient Active) | 330（14.8） | 276（12.4） | 241（10.8） | 847（37.9） |
| Total | 739（33.1） | 747（33.5） | 746（33.4） | 2232（100） |

**eFigure1. Dose-Response Association Between lnCAR and all-cause Mortality Among US Cancer Survivors**

1. **All**


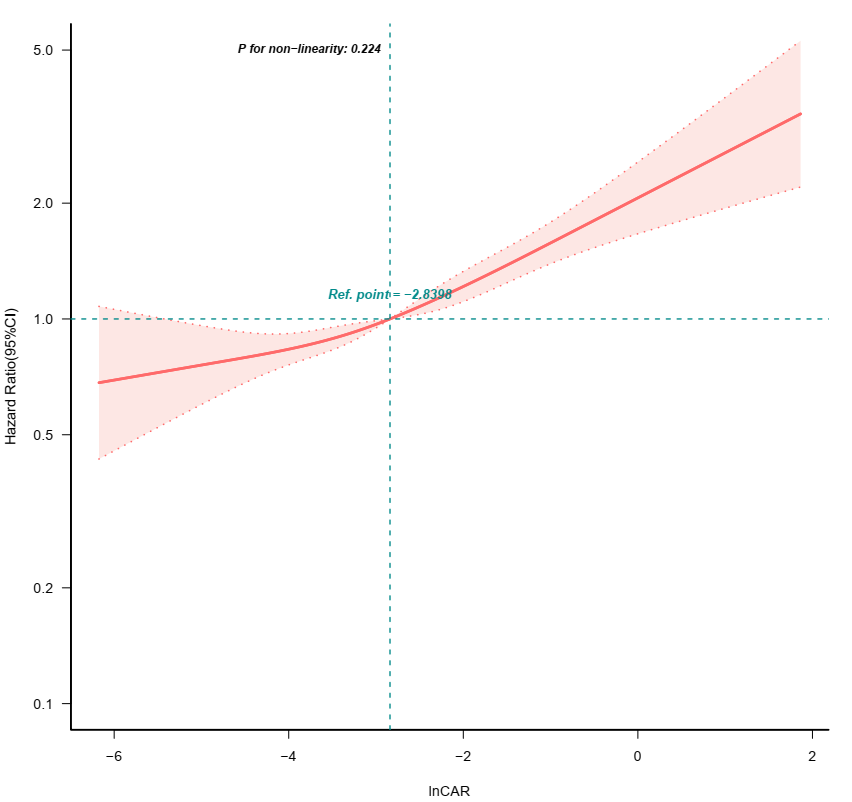


B **Insufficient active/inactive**


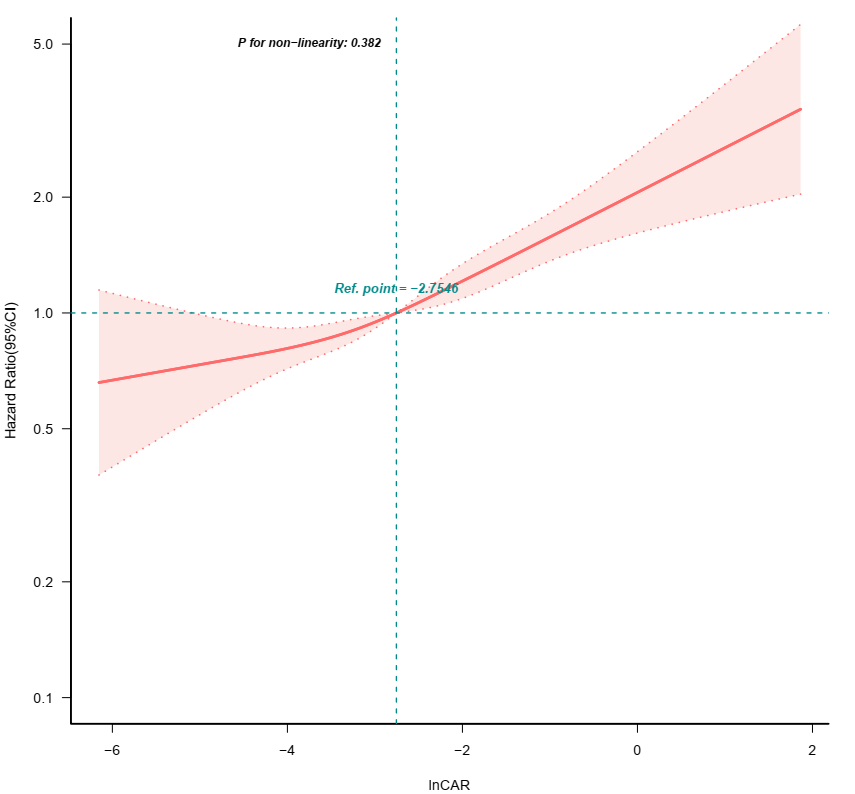


Data on lnCAR was fitted using a linear model (P <.001) with results reported as hazard ratios (IndianRed lines) and 95% CIs (shaded areas). Adjusted for age (years), sex (male or female), race/ethnicity (non-Hispanic white, non-Hispanic black, Hispanic, and other), education attainment (less than high school, high school graduate, above high school), family poverty ratio (<1.30, 1.30-3.49, or ≥3.5), body mass index (BMI; calculated as weight in kilograms divided by height in meters squared) (<25, 25-29.9, and ≥30), smoking status (never, former, and current), alcohol use (never, former, and current), Healthy Eating Index-2015, hypertension (yes or no), history of diabetes (yes or no), history of CVD (yes or no).

| **eTable 3. Association of CAR and Physical Activity Level with All-Cause Mortality Among US Cancer Survivors ≥40 years by Sex and Obesity-Related Cancer, NHANES 1999-2010** | | | | |
| --- | --- | --- | --- | --- |
| **Subgroup** | **Death/No.** | **Hazard Ratio (95% CI)** | |  |
|  |  | Non-adjusted Model | Model I^a^ | Model II^b^ |
| **Male** |  |  |  |  |
| CAR |  |  |  |  |
| Low | 211/423 | 1 [reference] | 1 [reference] | 1 [reference] |
| Medium | 223/369 | 1.29 (1.07~1.55) | 1.23 (1.01~1.48) | 1.18 (0.97~1.43) |
| High | 239/330 | 2.05 (1.7~2.47) | 1.84 (1.53~2.22) | 1.81 (1.49~2.19) |
| per SD increase |  | 1.19 (1.13~1.26) | 1.15 (1.09~1.21) | 1.13 (1.07~1.2) |
| Physical Activity Level |  |  |  |  |
| No PA (Inactive) | 249/324 | 1 [reference] | 1 [reference] | 1 [reference] |
| 0-<600 MET minutes/week PA  (Insufficient Active) | 176/309 | 0.44 (0.36~0.53) | 0.64 (0.52~0.78) | 0.67 (0.55~0.82) |
| ≥600 MET minutes/week PA  (Active) | 248/489 | 0.45 (0.38~0.54) | 0.58 (0.48~0.70) | 0.63 (0.52~0.77) |
| **Female** |  |  |  |  |
| CAR |  |  |  |  |
| Low | 118/316 | 1 [reference] | 1 [reference] | 1 [reference] |
| Medium | 184/378 | 1.41 (1.12~1.77) | 1.22 (0.97~1.54) | 1.24 (0.98~1.58) |
| High | 199/416 | 1.35 (1.07~1.69) | 1.47 (1.16~1.86) | 1.43 (1.11~1.82) |
| per SD increase |  | 1.13 (1.06~1.21) | 1.13 (1.07~1.19) | 1.11 (1.05~1.18) |
| Physical Activity Level |  |  |  |  |
| No PA (Inactive) | 259/435 | 1 [reference] | 1 [reference] | 1 [reference] |
| 0-<600 MET minutes/week PA  (Insufficient Active) | 142/317 | 0.56 (0.45~0.68) | 0.62 (0.50~0.76) | 0.63 (0.51~0.78) |
| ≥600 MET minutes/week PA  (Active) | 100/358 | 0.37 (0.29~0.46) | 0.47 (0.37~0.6) | 0.50 (0.39~0.63) |
| **Obesity-Related Cancer Survivor** |  |  |  |  |
| CAR |  |  |  |  |
| Low | 90/205 | 1 [reference] | 1 [reference] | 1 [reference] |
| Medium | 143/251 | 1.46 (1.12~1.90) | 1.27 (0.97~1.66) | 1.44 (1.09~1.90) |
| High | 146/260 | 1.45 (1.12~1.89) | 1.60(1.22~2.10) | 1.54 (1.17~2.04) |
| per SD increase |  | 1.44 (1.06~1.95) | 1.42 (1.09~1.85) | 1.30 (0.98~1.73) |
| Physical Activity Level |  |  |  |  |
| No PA (Inactive) | 181/293 | 1 [reference] | 1 [reference] | 1 [reference] |
| 0-<600 MET minutes/week PA  (Insufficient Active) | 103/190 | 0.68 (0.54~0.87) | 0.62 (0.48~0.79) | 0.61 (0.47~0.79) |
| ≥600 MET minutes/week PA  (Active) | 95/233 | 0.53 (0.41~0.67) | 0.54 (0.41~0.70) | 0.55 (0.42~0.72) |
| **Non-Obesity-Related Cancer Survivor** | |  |  |  |
| CAR |  |  |  |  |
| Low | 236/531 | 1 [reference] | 1 [reference] | 1 [reference] |
| Medium | 263/495 | 1.24(1.04~1.47) | 1.18 (0.99~1.41) | 1.12 (0.93~1.34) |
| High | 289/482 | 1.59 (1.34~1.89) | 1.69 (1.42~2.01) | 1.64 (1.37~1.96) |
| per SD increase |  | 1.70 (1.46~1.97) | 1.53 (1.33~1.76) | 1.48 (1.28~1.71) |
| Physical Activity Level |  |  |  |  |
| No PA (Inactive) | 324/463 | 1 [reference] | 1 [reference] | 1 [reference] |
| 0-<600 MET minutes/week PA  (Insufficient Active) | 213/434 | 0.45 (0.38~0.54) | 0.64 (0.53~0.77) | 0.65 (0.54~0.79) |
| ≥600 MET minutes/week PA  (Active) | 251/611 | 0.44 (0.37~0.52) | 0.54 (0.46~0.65) | 0.60(0.50~0.71) |
| ^a^ Multivariable model additionally adjusted for age (years), sex (male or female), race/ethnicity (non-Hispanic white, non-Hispanic black, Hispanic, and other), marital status (married or living with partner, widowed or divorced or separated or never married), education attainment (less than high school, high school graduate, above high school), family poverty ratio (<1.30, 1.30-3.49, or ≥3.5). | | | | |
| ^b^ Additionally adjusted for body mass index (BMI; calculated as weight in kilograms divided by height in meters squared) (<24.9, 25-29.9, and ≥30), smoking status (never, former, and current), alcohol use (never, former, and current),  and Healthy Eating Index-2015, Hypertension (yes or no), history of diabetes (yes or no), history of CVD (yes or no). | | | | |

| **eTable 4. Joint Association of CAR and Physical Activity Level With All-Cause Mortality Among US Cancer Survivors ≥40 years By Sex and Obesity-Related Cancer, NHANES 1999-2010** | | | | | |
| --- | --- | --- | --- | --- | --- |
| **Subgroup** | CAR | **Death/No.** | **Hazard Ratio (95% CI)** | |  |
|  |  |  | Non-adjusted Model | Model I^a^ | Model II^b^ |
| **Male** |  |  |  |  |  |
| ≥600 MET minutes/week PA  (Physically Active) | Low | 97/213 | 0.41 (0.32~0.53) | 0.45 (0.35~0.58) | 0.49 (0.37~0.63) |
|  | Medium | 77/163 | 0.43 (0.33~0.56) | 0.48 (0.36~0.63) | 0.50 (0.38~0.66) |
|  | High | 74/113 | 0.78 (0.59~1.02) | 0.77 (0.58~1.01) | 0.79 (0.6~1.05) |
| <600 MET minutes/week PA (Inactive/Insufficient Active) | Low | 114/210 | 0.48 (0.38~0.61) | 0.54 (0.42~0.69) | 0.54 (0.42~0.69) |
|  | Medium | 146/206 | 0.71 (0.57~0.88) | 0.70 (0.56~0.88) | 0.69 (0.55~0.86) |
|  | High | 165/217 | 1 [reference] | 1 [reference] | 1 [reference] |
| **Female** |  |  |  |  |  |
| ≥600 MET minutes/week PA  (Physically Active) | Low | 28/117 | 0.36 (0.24~0.54) | 0.39 (0.26~0.59) | 0.42 (0.28~0.64) |
|  | Medium | 38/113 | 0.54 (0.38~0.77) | 0.54 (0.38~0.78) | 0.56 (0.38~0.81) |
|  | High | 34/128 | 0.40 (0.27~0.57) | 0.49 (0.34~0.71) | 0.52 (0.35~0.76) |
| <600 MET minutes/week PA (Inactive/Insufficient Active) | Low | 90/199 | 0.73 (0.57~0.95) | 0.66 (0.51~0.86) | 0.67 (0.51~0.89) |
|  | Medium | 146/265 | 0.96 (0.77~1.2) | 0.76 (0.6~0.95) | 0.81 (0.64~1.02) |
|  | High | 165/288 | 1 [reference] | 1 [reference] | 1 [reference] |
| **Obesity-Related Cancer Survivor** | |  |  |  |  |
| ≥600 MET minutes/week PA  (Physically Active) | Low | 30/81 | 0.46 (0.31~0.69) | 0.42 (0.27~0.64) | 0.46 (0.30~0.71) |
|  | Medium | 36/76 | 0.67 (0.46~0.97) | 0.58 (0.39~0.85) | 0.65 (0.44~0.97) |
|  | High | 29/76 | 0.52 (0.35~0.78) | 0.51 (0.34~0.78) | 0.52 (0.34~0.80) |
| <600 MET minutes/week PA (Inactive/Insufficient Active) | Low | 60/124 | 0.67 (0.49~0.91) | 0.58 (0.42~0.80) | 0.57 (0.41~0.80) |
|  | Medium | 107/175 | 0.93 (0.72~1.21) | 0.70 (0.53~0.92) | 0.83 (0.62~1.11) |
|  | High | 117/184 | 1 [reference] | 1 [reference] | 1 [reference] |
| **Non-Obesity-Related Cancer Survivor** | |  |  |  |  |
| ≥600 MET minutes/week PA  (Physically Active) | Low | 94/248 | 0.47 (0.37~0.6) | 0.44 (0.35~0.57) | 0.49 (0.38~0.63) |
|  | Medium | 78/199 | 0.48(0.37~0.62) | 0.47 (0.36~0.61) | 0.49 (0.37~0.64) |
|  | High | 79/164 | 0.65 (0.50~0.85) | 0.71 (0.55~0.93) | 0.76 (0.58~0.99) |
| <600 MET minutes/week PA (Inactive/Insufficient Active) | Low | 142/283 | 0.61 (0.50~0.76) | 0.60 (0.49~0.75) | 0.60 (0.48~0.75) |
|  | Medium | 185/296 | 0.82 (0.68~1.0) | 0.73 (0.60~0.89) | 0.70 (0.58~0.86) |
|  | High | 210/318 | 1 [reference] | 1 [reference] | 1 [reference] |
| ^a^ Multivariable model additionally adjusted for sex (male or female), race/ethnicity (non-Hispanic white, non-Hispanic black, Hispanic, and other), marital status (married or living with partner, widowed or divorced or separated or never married), education attainment (less than high school, high school graduate, above high school), family poverty ratio (<1.30, 1.30-3.49, or ≥3.5). | | | | | |
| ^b^ Additionally adjusted for body mass index (BMI; calculated as weight in kilograms divided by height in meters squared) (<25, 25-29.9, and ≥30), smoking status (never, former, and current), alcohol use (never, former, and current), and Healthy Eating Index-2015，hypertension (yes or no), history of diabetes (yes or no), history of CVD (yes or no). | | | | | |

| **eTable 5. Association between C-reactive protein to albumin ratio and risk of death in multiple regression model(sensitivity analysis, excluding deaths that occurred during the first 2-year follow-up,n=2093).** | | | | | | | |
| --- | --- | --- | --- | --- | --- | --- | --- |
| **Outcome** | **Deaths /no.** | **Non-adjusted Model** | | **Model I^a^** | | **Model II^b^** | |
|  |  | **HR (95% CI)** | **P-value** | **HR (95% CI)** | **P-value** | **HR (95% CI)** | **P-value** |
| **Cancer Mortality** |  |  |  |  |  |  |  |
| CAR |  |  |  |  |  |  |  |
| Low | 73/711 | 1 [reference] |  | 1 [reference] |  | 1 [reference] |  |
| Medium | 100/718 | 1.44 (1.07~1.95) | 0.017 | 1.42 (1.05~1.92) | 0.024 | 1.31 (0.96~1.78) | 0.089 |
| High | 104/664 | 1.66 (1.23~2.23) | 0.001 | 1.8 (1.32~2.44) | <0.001 | 1.77 (1.29~2.42) | <0.001 |
| Physical Activity Level |  |  |  |  |  |  |  |
| No PA (Inactive) | 101/671 | 1 [reference] |  | 1 [reference] |  | 1 [reference] |  |
| 0-<600 MET minutes/week PA  (Insufficient Active) | 82/601 | 0.69 (0.51~0.92) | 0.013 | 0.78 (0.58~1.05) | 0.105 | 0.78 (0.58~1.06) | 0.114 |
| ≥600 MET minutes/week PA  (Active) | 94/821 | 0.63 (0.48~0.84) | 0.001 | 0.68 (0.51~0.91) | 0.01 | 0.71 (0.53~0.96) | 0.028 |
| **All-Cause Mortality** |  |  |  |  |  |  |  |
| CAR |  |  |  |  |  |  |  |
| Low | 301/711 | 1 [reference] |  | 1 [reference] |  | 1 [reference] |  |
| Medium | 378/718 | 1.33 (1.14~1.55) | <0.001 | 1.26 (1.08~1.46) | 0.003 | 1.23 (1.06~1.44) | 0.008 |
| High | 356/664 | 1.38 (1.19~1.61) | <0.001 | 1.51 (1.29~1.76) | <0.001 | 1.47 (1.25~1.72) | <0.001 |
| Physical Activity Level |  |  |  |  |  |  |  |
| No PA (Inactive) | 420/671 | 1 [reference] |  | 1 [reference] |  | 1 [reference] |  |
| 0-<600 MET minutes/week PA  (Insufficient Active) | 293/601 | 0.56 (0.48~0.65) | <0.001 | 0.67 (0.58~0.79) | <0.001 | 0.69 (0.59~0.81) | <0.001 |
| ≥600 MET minutes/week PA  (Active) | 322/821 | 0.51 (0.44~0.59) | <0.001 | 0.58 (0.50~0.68) | <0.001 | 0.64 (0.54~0.75) | <0.001 |
| ^a^ Multivariable model additionally adjusted for sex (male or female), race/ethnicity (non-Hispanic white, non-Hispanic black, Hispanic, and other), marital status (married or living with partner, widowed or divorced or separated or never married), education attainment (less than high school, high school graduate, above high school), family poverty ratio (<1.30, 1.30-3.49, or ≥3.5). | | | | | | | |
| ^b^ Additionally adjusted for body mass index (BMI; calculated as weight in kilograms divided by height in meters squared) (<25, 25-29.9, and ≥30), smoking status (never, former, and current), alcohol use (never, former, and current), and Healthy Eating Index-2015，hypertension (yes or no), history of diabetes (yes or no), history of CVD (yes or no). | | | | | | | |

| **eTable 6. Joint association between C-reactive protein to albumin ratio and Physical activity with all-cause and cancer mortality among US cancer survivors age 40 years or older, NHANES, 1999 to 2010.(sensitivity analysis, excluding deaths that occurred during the first 2-year follow-up,n=2093).** | | | | | | | | |
| --- | --- | --- | --- | --- | --- | --- | --- | --- |
| **outcome** |  | |  | | **Hazard ratio (95 % CI)** | | | |
|  | **Physical activity** | | **Death/No.** | | **Non-adjusted Model** | **Model I^a^** | | **Model II^b^** |
| **Cancer Mortality** |  | |  | |  |  | |  |
| CAR(low) | No PA (Inactive) | | 19/172 | | 0.69 (0.40~1.18) | 0.6 (0.35~1.04) | | 0.64 (0.37~1.11) |
|  | 0-<600 MET minutes/week PA  (Insufficient Active) | | 25/213 | | 0.55 (0.34~0.90) | 0.58 (0.35~0.96) | | 0.59 (0.35~0.99) |
|  | ≥600 MET minutes/week PA  (Active) | | 29/326 | | 0.48 (0.30~0.76) | 0.44 (0.27~0.71) | | 0.46 (0.28~0.76) |
| CAR(medium) | No PA (Inactive) | | 40/225 | | 1.19 (0.77~1.84) | 1.05 (0.68~1.62) | | 0.97 (0.63~1.52) |
|  | 0-<600 MET minutes/week PA  (Insufficient Active) | | 24/222 | | 0.55 (0.33~0.91) | 0.54 (0.32~0.90) | | 0.51 (0.31~0.86) |
|  | ≥600 MET minutes/week PA  (Active) | | 36/271 | | 0.72 (0.46~1.13) | 0.68 (0.43~1.08) | | 0.67 (0.42~1.07) |
| CAR(high) | No PA (Inactive) | | 42/274 | | 1 [reference] | 1 [reference] | | 1 [reference] |
|  | 0-<600 MET minutes/week PA  (Insufficient Active) | | 33/166 | | 1.02 (0.65~1.61) | 1.08 (0.68~1.72) | | 1.1 (0.69~1.76) |
|  | ≥600 MET minutes/week PA  (Active) | | 29/224 | | 0.71 (0.44~1.14) | 0.78 (0.48~1.26) | | 0.82 (0.51~1.33) |
| **All-Cause Mortality** |  | |  | |  |  | |  |
| CAR(low) | No PA (Inactive) | | 94/172 | | 0.81 (0.63~1.04) | 0.65 (0.50~0.84) | | 0.65 (0.50~0.84) |
|  | 0-<600 MET minutes/week PA  (Insufficient Active) | | 86/213 | | 0.42 (0.33~0.55) | 0.47 (0.36~0.61) | | 0.45 (0.35~0.6) |
|  | ≥600 MET minutes/week PA  (Active) | | 121/326 | | 0.46 (0.37~0.59) | 0.43 (0.34~0.55) | | 0.47 (0.37~0.6) |
| CAR(medium) | No PA (Inactive) | | 148/225 | | 1.06 (0.85~1.32) | 0.84 (0.67~1.05) | | 0.81 (0.65~1.01) |
|  | 0-<600 MET minutes/week PA  (Insufficient Active) | | 120/222 | | 0.62 (0.49~0.78) | 0.58 (0.46~0.74) | | 0.61 (0.48~0.77) |
|  | ≥600 MET minutes/week PA  (Active) | | 110/271 | | 0.52 (0.41~0.65) | 0.49 (0.38~0.63) | | 0.51 (0.40~0.66) |
| CAR(high) | No PA (Inactive) | 178/274 | | 1 [reference] | | | 1 [reference] | 1 [reference] |
|  | 0-<600 MET minutes/week PA  (Insufficient Active) | 87/166 | | 0.61 (0.47~0.79) | | | 0.67 (0.51~0.86) | 0.66 (0.51~0.86) |
|  | ≥600 MET minutes/week PA  (Active) | 91/224 | | 0.52 (0.40~0.67) | | | 0.57 (0.44~0.74) | 0.61 (0.47~0.79) |
| a Multivariable model additionally adjusted for sex (male or female), race/ethnicity (non-Hispanic white, non-Hispanic black, Hispanic, and other), marital status (married or living with partner, widowed or divorced or separated or never married), education attainment (less than high school, high school graduate, above high school), family poverty ratio (<1.30, 1.30-3.49, or ≥3.5). | | | | | | | | |
| b Additionally adjusted for body mass index (BMI; calculated as weight in kilograms divided by height in meters squared) (<25, 25-29.9, and ≥30), smoking status (never, former, and current), alcohol use (never, former, and current), and Healthy Eating Index-2015，hypertension (yes or no), history of diabetes (yes or no), history of CVD (yes or no). | | | | | | | | |

**eFigure2. Flowchart of patient selection.**


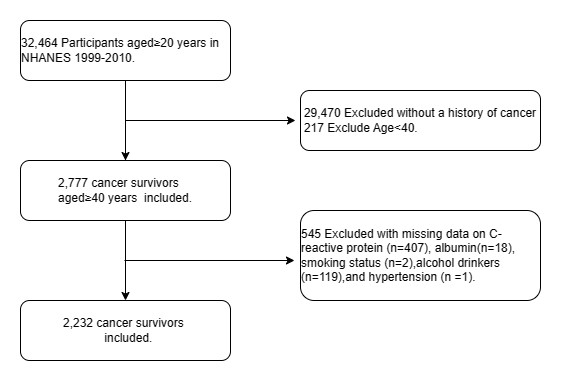

Supplement: Supplementary file 1 — Supplementary Material 1 [file 11556_2024_361_MOESM1_ESM.docx]
